# Influence of artificial intelligence on the work design of emergency department clinicians a systematic literature review

**DOI:** 10.1186/s12913-022-08070-7

**Published:** 2022-05-18

**Authors:** Albert Boonstra, Mente Laven

**Affiliations:** Faculty of Economics and Business, University of Groningen, Groningen, The Netherlands

**Keywords:** Artificial Intelligence, Clinicians, Emergency department, Machine Learning, Work design

## Abstract

**Objective:**

This systematic literature review aims to demonstrate how Artificial Intelligence (AI) is currently used in emergency departments (ED) and how it alters the work design of ED clinicians. AI is still new and unknown to many healthcare professionals in emergency care, leading to unfamiliarity with its capabilities.

**Method:**

Various criteria were used to establish the suitability of the articles to answer the research question. This study was based on 34 selected peer-reviewed papers on the use of Artificial Intelligence (AI) in the Emergency Department (ED), published in the last five years. Drawing on the Preferred Reporting Items for Systematic Reviews and Meta-Analyses (PRISMA) guidelines, all articles were scanned, read full-text, and analyzed afterward.

**Results:**

The majority of the AI applications consisted of AI-based tools to aid with clinical decisions and to relieve overcrowded EDs of their burden. AI support was mostly offered during triage, the moment that sets the patient trajectory. There is ample evidence that AI-based applications could improve the clinical decision-making process.

**Conclusion:**

The use of AI in EDs is still in its nascent stages. Many studies focus on the question of whether AI has clinical utility, such as decision support, improving resource allocation, reducing diagnostic errors, and promoting proactivity. Some studies suggest that AI-based tools essentially have the ability to outperform human skills. However, it is evident from the literature that current technology does not have the aims or power to do so. Nevertheless, AI-based tools can impact clinician work design in the ED by providing support with clinical decisions, which could ultimately help alleviate a portion of the increasing clinical burden.

**Supplementary Information:**

The online version contains supplementary material available at 10.1186/s12913-022-08070-7.

## Background

Over the past years, the need for a higher quality of care has increased significantly. Healthcare faces significant challenges of a “rising burden of illness, multimorbidity and disability driven by aging and epidemiological transition, greater demand for health services, higher societal expectations, and increasing health expenditures” [1]. To respond to these challenges, healthcare must continue to improve its productivity and efficiency, which raises the question of whether healthcare professionals' expectations to deliver good healthcare might still be within human capabilities [2].

Healthcare providers globally recognize that part of the solution to these challenges is to embed artificial intelligence (AI) into their work processes [3]. AI is machine-learned intelligence instead of the natural intelligence humans or animals display. It is the concept of computer systems performing tasks that usually demands human knowledge [4]. AI applications aim to comprehend and develop electronic methods that embed intelligence properties [1].

AI is increasingly used in healthcare as it can work as a catalyst to overcome significant challenges of health systems. While “AI” is often understood as either complex and all-encompassing or vague, it comes down to a computer that simulates human intelligence by learning to make deductions when fed new data [3]. There are subdivisions in kinds of AI technology, and one of them is machine learning [4]. ML can improve algorithms by recognizing patterns in large numbers of data and can make calculations or predictions using statistical approaches [4, 5]. For example, a prediction model using ML can recognize heart rate and blood pressure patterns, which can help detect sepsis at an earlier stage, significantly improving patient outcomes [4].

One particular strength of AI, the speed with which it can make inferences, makes it relevant for emergency medicine. In emergency departments (EDs), a fast interpretation of clinical data to categorize the severity of patients’ conditions is of great importance [3]. One of the current standard methods to achieve this is the Emergency Severity Index (ESI) assistance, which helps triage patients at high speed [3]. However, this method relies heavily on subjective data, which makes it prone to errors [3]. This makes AI even more helpful, as it has shown high accuracy in addition to speed [6].

Although some argue that AI might eventually take over some of the work of emergency personnel, such as radiologists, the evidence currently shows that AI can significantly improve the quality and speed of emergency medicine [7, 8]. In emergency medicine, speed is essential, so a computer's quick “brain” could be used in such an environment [9]. Nevertheless, because the thinking ability of AI exceeds human capacity and pace, AI can alter the role of the emergency physician. The less complex tasks, such as interpreting images, could be unraveled with AI. At the same time, physicians focus on the more challenging aspects of the job, such as communication with professionals and patients [10]. While research on AI’s clinical utility is increasing, no studies assess the influence of AI clinical decision support tools on ED clinician behavior and patient flow [11].

To advance our understanding of AI use in emergency medicine, this paper provides a systematic literature review to examine the effects of AI on the work design of emergency clinicians. This study mainly focuses on recent research regarding 1) the purposes of AI use in emergency care, 2) how AI is used in EDs, 3) the effects of AI on ED’s functioning, and 4) the effects on the work design ED clinicians. In doing so, we examine the following research question: *According to recent research, why and how is Artificial Intelligence currently used in Emergency Departments, and how does it alter the work design of emergency clinicians?*

By addressing this research question, we complement related studies on AI in emergency care [12, 13] with a distinct focus on why and how AI is used in emergency care and its effects on the work design of clinicians.

## Method

### Search strategies

The search for relevant articles was done through SmartCat, Web of Science, and PubMed on the 21^st^ of April 2021. These databases generated the most significant number of relevant articles. The following search terms were used: 1) Artificial Intelligence AND Emergency Medicine/Department, 2) Machine Learning AND Emergency Medicine/Department/Room. Additional articles were retrieved through a backtrack-searching method. This method involves scanning the reference list of other articles on the research topic. Backtracking was done in four academic papers used earlier in this research. All articles focus on the use of artificial intelligence in the emergency department.

**Fig. 1:**
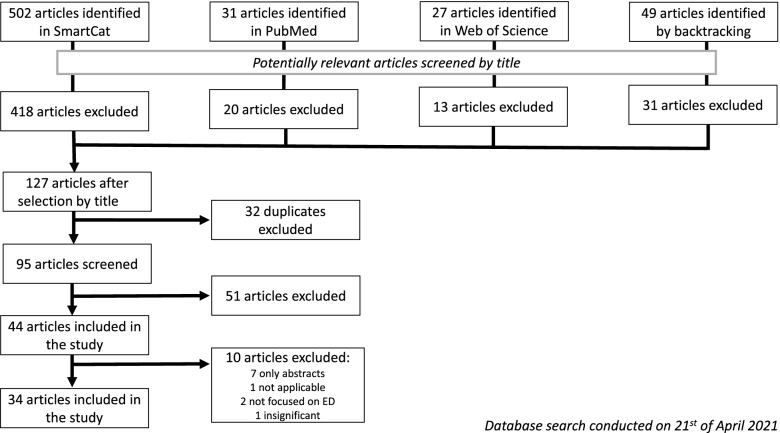
Flow of the article selection through the different phases of a systematic review

Figure 1 shows a flowchart of the search strategy for relevant articles. This strategy includes all the initial search results [14] to narrow the search eventually. The selection process consists of multiple stages that are included in the flowchart. The first stage contains an initial search with keywords; the second step lets the articles through the sieve to exclude unsuitable papers. One must, for example, exclude duplicate articles or articles with misleading titles. Finally, the remaining articles are read in their entirety and incorporated into the review when suitable [14].

The literature was selected based on its year of publication, which should not be before 2017. Information technology moves incredibly fast, which means that articles older than five years will possibly be outdated. Furthermore, the papers need to be peer-reviewed to ensure their quality. Additionally, the articles must be written in English to make them more widely discernable. Moreover, papers should be unique and available in full text, and other systematic literature reviews were excluded from the search. The search in SmartCat generated a list of 502 articles, of which 418 papers were excluded because they were unrelated to the research question. In PubMed, the search resulted in a list of 31 articles, of which 20 articles were irrelevant, and in Web of Science the search resulted in 27 articles, of which 13 were irrelevant. Eventually, 106 articles were initially included during the Boolean search (Table 1). In that phase of the article selection, we did not yet use duplication as an exclusion criterion.

**Table 1:** Selected studies (see additional file 4 for more detail)

Article nr	The main aim of the study
[11]	To reduce cognitive load on clinicians by predicting the risk for admission
[15]	To reduce mortality by predicting the risk for (severe) sepsis in the ED
[16]	To help physicians by predicting the need for hospitalization
[17]	To help streamline crowded EDs by developing an AI tool that could remove the need for an expert emergency medicine physician during triage
[18]	To enhance ED triage systems by predicting mortality risk and risk for cardiac arrest
[19]	To prevent overcrowding of EDs by predicting future ED visits
[20]	To reduce ED morbidity and mortality by predicting the disposition of asthma and COPD exacerbation after triage
[21]	To increase physician satisfaction and reduce physician burnout by improving the efficiency and quality of structured data
[22]	To reduce/prevent overcrowding of EDs and improve patient care by predicting the need for hospitalization
[23]	To reduce ED morbidity and mortality costs by predicting risk for sepsis at triage and by implementing protocolized care
[24]	To reduce the length of stay (LOS) in ED by predicting clinical ordering at triage
[25]	To reduce/prevent overcrowding of EDs by predicting the risk for cardiac arrest in ED
[26]	To reduce ED morbidity and mortality and overcrowding of EDs by predicting triage levels for patients with suspected cardiovascular disease (CVD)
[27]	To cope with the increasing demand for clinical care in EDs by predicting septic shock at triage
[28]	To alleviate overburdened EDs and increase patients’ throughput by identifying patients’ need for a head CT scan at triage
[29]	To alleviate overburdened EDs by improving patient categorization by predicting ED mortality
[30]	To improve patients’ throughput in EDs by identifying severe thorax injury
[31]	To reduce overcrowding of EDs by predicting patient waiting times
[32]	To reduce overcrowding of EDs by developing an e-triage system
[33]	To improve patient outcomes and reduce adverse effects by identifying patients at risk for acute kidney failure
[34]	To prevent adverse outcomes by predicting/identifying the geriatric need for hospitalization
[35]	To improve patient outcomes by identifying scaphoid fractures
[36]	To improve patient outcomes by predicting patient waiting times
[37]	To cope with overcrowding of EDs through predicting critical care and hospitalization outcomes at triage
[38]	To improve patient outcomes by linking prehospital records to hospital records
[39]	To safely reduce hospital admissions by predicting risk for 30-day adverse severe events
[40]	To improve patient outcomes and enhance physician ability by identifying ECG outcomes
[41]	To increase patient throughput in crowded EDs by predicting patient disposition during triage
[42]	To reduce diagnostic errors (and costs & overutilization of resources) by predicting/identifying urinary tract infections (UTIs) early
[43]	To improve healthcare delivery by predicting future hospital demand
[44]	To improve healthcare provider wellbeing and preserve patient safety by predicting clinician workload
[45]	To cope with overcrowding of EDs by predicting adverse clinical outcomes at tirage
[46]	To improve patient outcomes by identifying septic shock at an early stage
[47]	To reduce diagnostic errors and excess costs by predicting and identifying severe cardiac events

Additional articles were retrieved by backtracking in four academic papers on artificial intelligence in emergency departments (Additional file 1). For this method, articles in the reference list with artificial intelligence, a synonym, and emergency department, or a synonym, were selected. This resulted in an additional list of 49 articles. After removing duplicates, 40 articles remained. Then, to maintain consistency, all articles published before 2017 were removed from this list, leading to a final result of 18 articles. After this step, the two lists were combined, and at this point, all duplicates were excluded. Twenty-nine articles had to be removed, and 95 remained on the list.

We screened these 95 articles to establish a better and more comprehensive understanding of their relevance to answering the research question. The focus during this screening was on removing excess reviews and whether the studies focused on AI in emergency departments. Lastly, studies that concluded a negative outcome, meaning that the AI-based application did not outperform conventional methods, were deemed irrelevant. It must be mentioned that such studies do contribute to the research field of AI in general, yet for answering our research question, they do not provide helpful information. In short, these studies do not have the potential to alter the work of ED clinicians. After the screening, 44 articles remained. After reading the abstracts, 51 articles were excluded, as they turned out to be irrelevant. Additional file 2 shows a numerical list of the articles accompanied by a color-coding scheme. This file shows the main themes of the papers based on the abstract, including the reasons for exclusion. At this point, the articles were not yet placed in alphabetical order but in order of retrieving.

## Data analysis

Drawing from the coding scheme (Additional file 2), 44 articles remained to be evaluated. This was done by reading the articles' full text. During this analysis, it was crucial that the effects on the work design of emergency clinicians could be identified. Essentially, this means differentiation of themes between the scope of the study and its impact on the work design of ED clinicians. Two main questions guided our analysis during this initial scanning:

- 1. Does the research address the influence of AI on the job design of ED physicians?
- 2. Does the AI in the research potentially replace doctors when applied intensively?

Additional file 3 shows a table with answers to these questions and motivations per text. Additionally, this table was used as a setup for the actual review later in the research. The numbers in this table are derived from the first round of abstract scanning, meaning that the numbers correspond. Seven were removed from the 44 articles scanned because only abstracts could be found. One was removed because the kind of AI used in the research was not applicable in a clinical setting, and another two were removed because the focus was not on the ED. Two studies [17, 35] followed a somewhat distinctive research method, as these used real physicians in their studies. Instead of merely using data comparisons – which was done in the remaining studies – these two studies focused on comparing human capabilities and AI. Although they are somewhat different than the other studies, they are eventually included in this review. The outcomes of these two studies are essential for the current discussion and are therefore included in the review. In the end, 34 articles remained.

The resulting 34 papers are organized by the research aim in Table 1. Additional file 4 provides more detailed information.

## Results

After analyzing the remaining 34 studies, the following are some of our general results. Most of the studies were conducted in either America (*n* = 15, 45.5%) or Asia (*n* = 11, 32.4%) and the majority (*n* = 19, 55.9%) of the studies used data either collected or available (from the EHR) at the stage of triage.

### The purposes of AI use in emergency care

To understand the influence of AI on the jobs of emergency clinicians, it is necessary to establish for what kind of purposes AI is implemented in the ED. Three themes emerged and are coded to show this clearly and concisely (Table 3). The themes represent the underlying problem that the studies aim to solve (1), how the studies aim to solve this problem (2), and the focus area of AI use (3). The codes are divided by the intended use types of AI in emergency departments. Essentially, all studies aim to help with providing better care. However, there are differences, and Table 2 shows the specific main findings on this topic.

**Table 2:** Purposes of AI in emergency departments

Code	The purpose of AI use	Studies addressing this purpose
**1 Underlying problem they aim to solve**		
** 1a**	To improve patient outcomes (including mortality, morbidity, and satisfaction)	15, 16, 20, 22–24, 26, 33–36, 38, 40, 43, 44, 46
** 1b**	To reduce or cope with overcrowded EDs	17, 19, 22, 25–32, 37, 41, 45
**2 The means through which the studies aimed to solve the overarching problem**		
** 2a**	To accurately predict future outcomes	11, 15, 16, 18, 19, 22–31, 34, 36, 37, 39, 41–45, 47
** 2b**	To accurately identify outcomes	28, 30, 33, 35, 40, 42, 46, 47
** 2c**	To reduce the need for a physician	17, 35
**3 The focus area of AI use**		
** 3a**	To improve ED triage in general or through the prediction or identification of serious or critical (adverse) outcomes	17, 18, 20–29, 32, 37, 41, 45
** 3b**	To assist clinicians with the prediction or identification of serious or critical (adverse) outcomes	15, 16, 30, 33, 39, 42, 46, 47
** 3c**	To assist in predicting or identifying non-critical (adverse) outcomes	11, 19, 31, 34–36, 38, 40, 43, 44

**1a:** Improving patient outcomes is a broad term, as it is an overarching term for many different things, such as fewer complications and reducing morbidity. To address the essence of healthcare, which is to provide good care to people. Most studies do not further define patient outcomes; however, some do. Included in patient outcomes are reducing morbidity and mortality, [15, 20, 23, 26] reducing the length of hospital stay [24], and improving patient satisfaction [36].

**1b:** Another frequently mentioned issue is that EDs are overcrowded. ED overcrowding results from factors, the most prominent being the longer living aging population [19]. In the US, for example, the number of ED visits has doubled during the last 20 years [37]. Reducing the number of visits would have several consequences for clinicians and patients, such as increasing the physician's time with a patient.

Together with reducing overcrowded EDs, AI in emergency care aims to better cope with overcrowding EDs in another way. Reducing the number of visits is, in some cases, not feasible. What is possible is to manage the number of patients that come into an ED. Coping with overcrowding EDs can be done in multiple ways, such as speeding up patient throughput [30] or increasing flow [41].

**2a:** To improve the management of overcrowded EDs, many studies focus on predicting the risk of certain complications and the future condition of patients. This can help doctors make decisions about making resources available to high-risk patients.

**2b:** The line between predicting and identifying is quite blurry, as predicting and early identification nearly share the same meaning. However, early identification means that the patient is already developing a particular outcome, while prediction can prevent the patient from developing it. Nevertheless, they often go hand in hand. It depends on how early the identification takes place. Very early identification of a particular outcome may, just as prediction, prevent a serious (adverse) event from happening.

**2c:** In addition to predicting and identifying future outcomes, two studies focus on these aspects in combination with reducing the need for a physician’s attention. Although only two studies focus on reducing the need for a physician’s attention, it is critical to address because it tackles whether human clinicians become replaceable at some point in the future.

**3a:** The prediction and identification of outcomes were most often made through triaging. Articles 21, 24, 32, and 41 focused on improving the ED triage system because ESI works sub-optimally. In addition, triage sets the course for ED care as it is the first moment at which patients can be categorized [32]. An example of a measure that can help to improve triaging is implementing an electronic triage system (e-triage) that predicts the probability of critical outcomes [32].

The remaining 14 studies aimed to improve ED triage by predicting or identifying specific serious or critical (adverse) outcomes. As there are many events of frequent occurrence in the ED, the list of critical (adverse) events to improve ED triage is diverse. The list of events from the literature contains acute abdominal pain [17], cardiac arrest [18, 25], exacerbation of asthma or COPD [20], sepsis or septic shock [23, 27], CVD [26], need for a head CT [28], clinical adverse events in general [45], hospitalization or discharge [22, 37], and mortality [18, 29).

**3b:** Not all critical outcomes are present during triage; thus, some literature centers around improving ED practices after triaging. Examples of this are identifying septic shock [15, 46] or predicting risk for a 30-day adverse event [39].

**3c:** Several studies aim to predict or identify severe but non-critical events such as the need for hospitalization or admission. Others seek to reduce the long waiting times often noted in EDs [31, 36]. Another example of a non-critical event can be found in [35], which focuses on wrist fractures.

### Influence of AI on work design in emergency units

After establishing for what kind of purposes AI is used, the influences on the work design of clinicians can be discussed. Results are shown in Table 3.

**Table 3:** Key Findings of influence of AI on emergency departments

Code	Key Findings: effects on work design	Studies addressing influences
4a	It can be used as a clinical decision support tool	11, 15, 16, 19, 20, 22–24, 26, 28–30, 32, 33, 37, 39, 42, 45–47
4b	It can improve healthcare delivery	19, 20, 25, 37
4c	It can alter management	15, 16, 38, 43
4d	It can improve resource allocation (including personnel)	15, 16, 24, 27, 31, 37, 41
4e	It can enhance (hospital) efficiency (including costs)	16, 19–21, 24, 28, 41, 43

**4a:** In most literature, predicting or identifying problems is achieved through an AI-based clinical decision support tool (CDST). CDSTs have been around for over a decade, and they can assist clinicians in making clinical decisions. These conventional CDSTs are often based on statistical predictions [11]. AI-based CDSTs could prove to be even more accurate than traditional CDSTs. The effect of this might, i.e., be an improvement in healthcare delivery.

**4b:** To elaborate, three studies conclude that AI-based tools can improve healthcare delivery. For example, [25] explains that their model for predicting cardiac arrest can reduce alarm fatigue and desensitization because the number of false alarms would decrease. As a result, this could result in advancements in healthcare.

**4c:** AI-based tools can alter ED management on a more centered and business scale. Although not often specified in the literature, an example might be a management device or model based on a clinician’s workload [44].

**4d:** Succeeding the improvement of healthcare delivery, AI-based tools can improve the resource allocation of EDs, including the allocation of personnel. For example, schedules can be adjusted according to the demand by predicting hospital demand [43].

**4e:** An enhanced resource allocation could also lead to increased efficiency. This can imply several things, and most studies that address efficiency do not specify what efficiency precisely means. However, one established form of efficiency is a reduction of costs [19].

### Influence of AI on work design of ED clinicians

In addition to the influences found on the general work design in the ED, there are influences on the work design for clinicians specifically (Table 4).

**Table 4:** Influence of AI on work design of ED clinicians

Code	Key Findings: influences on clinicians	Studies addressing influences
5a	Reduces workload/burden on clinicians	21, 26, 42, 44
5b	Improves and reduces variation in decision making	16, 17, 20, 25, 26, 29, 32, 39, 40, 45
5c	Reduces (diagnostic) errors	26, 29
5d	Changes reactive handling to proactive handling	11, 24, 31, 34
5e	Replaces physicians	17, 28, 35, 41

**5a:** With a growing demand for healthcare, the cognitive workload on clinicians is rising, and by using an AI-based tool as support, this burden can be reduced. This way, clinicians can refocus on clinical care again [26].

**5b**: As part of this refocus on clinical care, the majority of the literature in this section mentions that using an AI-based tool can help improve the decision-making process for clinicians and reduce differences among clinicians. For example, an AI-based CDST can warn the clinician when it recognizes abnormalities that are hard to detect and thus often missed [40].

**5c:** Although some literature addresses non-diagnostic errors, it was not specified what reducing non-diagnostic errors could entail. Correspondingly, two studies explain that by implementing an AI-based tool, (diagnostic) errors can be reduced. These errors can be reduced by preventing over-or under-triaging [29].

**5d:** Several studies also mention that implementing AI in the ED can help shift from reactive handling to proactive handling. For example, when there is a predicted increase in waiting times, doctors on-call can be paged timely [31].

**5e**: Conversely, several studies designed an AI-based tool that can assist and, at times, partly replace a physician. Two of the studies in this category concluded that their AI-based tool could replace a physician. However, their specific intent was not to do so [28, 41].

## Discussion

**Fig. 2:**
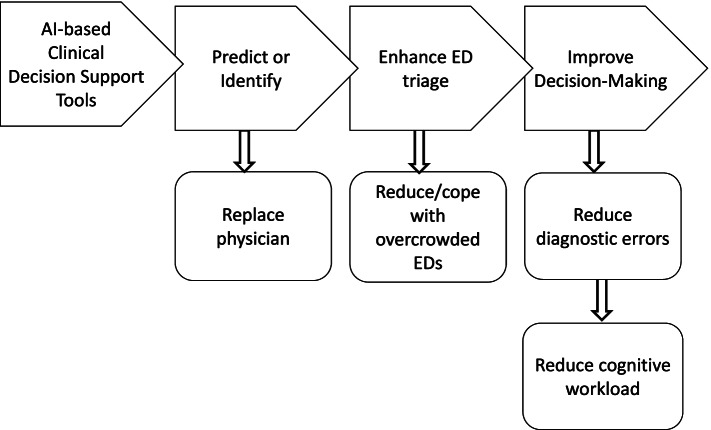
Model of types of AI use in ED and possible consequences

To answer the research question, the key findings of the literature will be discussed in this section. Furthermore, this section will discuss the gap in the literature, propose recommendations for future research, and indicate the limitations of this study. Figure 2 graphically visually displays the main findings of this study. This model depicts the most prominent causational processes during the research, accompanied by other (future) effects.

The literature shows that AI-based tools in the ED aim to improve patient outcomes and the work of clinicians. Patients will receive better treatment when clinicians can adequately perform their work, and it also works in the opposite direction. Thus, when the quality of care increases, e.g., a decrease in mortality, clinicians could gain more fulfillment from their job.

The majority of the literature also demonstrated that AI is used to either reduce or cope with overcrowded EDs through triage. These problems often contained implementing an AI-based CDST that could predict or identify future outcomes. According to [33], implementing AI-based CDSTs is essential because clinicians treat more patients. The workload of ED and its clinicians is rising while it is physically impossible to keep up with de demand [28]. For example, AI-based CDSTs can accelerate treatment and identify patients at high risk for mortality and under-triaged [29]. Most CDSTs aim to improve ED triaging with prediction or identification tools, as triaging is the first-moment patients can be categorized. Therefore, it sets the course for ED care [32].

AI-based CDSTs aim to support clinical decision-making. They do not seek to substitute clinical judgment [29]. The effects of using AI-based CDSTs are numerous. Still, the essential effects for clinicians entail that it can improve the decision-making process while reducing diagnostic errors and the cognitive workload. Although not multiple, the literature shows some evidence of AI-based tools being able to replace physicians. However, the studies aiming to create a device capable of replacing physicians do not seek to replace them. [35] argues that their tool does not outperform all kinds of physicians. For example, it can be helpful for hospitals that do not have a specific type of specialist available to make informed clinical decisions. [17] explains that their tool can only partially replace an expert physician and can be used in overcrowding situations to accelerate decision-making. Reducing the need for a physician does not necessarily mean that humans become unnecessary. It can add to better care when hospitals do not have enough (human) resources [17]. Nevertheless, because these AI-based CDSTs can continue to learn, they will most likely have the ability to outperform physicians in the future [35]. Although several studies explicitly mention that the AI-based CDSTs are not meant to replace clinical judgment, sometimes this still occurs unintentionally [28, 41]. This means that eventually, some jobs can become redundant. However, it will take years before technology reaches that point, and only time will tell what will happen when it does.

Notwithstanding the results, this research has several limitations. Firstly, the studies considered in this review do not address the implications that AI-based tools can have on the work design of clinicians. These studies mainly emphasized whether AI had clinical utility and what it can do for healthcare in general, making it difficult to be specific about work design. Secondly, this paper does not address the different kinds of AI-based tools used in the studies, although shown in the literature. Implementing this information could have led to more specific results; however, it was beyond the scope of this research. Thirdly, most studies explain that there is currently an implementation barrier because research on AI use in the ED is in its early stages. If a tool works for a specific patient population, it does not imply that it works for all patients. However, a strength of ML is that it is easy to retrain [43] and that it always keeps learning [40]. Finally, we acknowledge that the studies selected in this review report primarily analyze the effective use of AI in emergency care. This can give the wrong impression that implementing AI and emergency care is straightforward. We encourage research that focuses on barriers to AI in emergency care and the causes of medical staff's resistance to AI applications. This allows us to understand better which AI applications and conditions promote and inhibit effective AI use.

## Conclusion

This systematic literature review has focused on how the use of AI in the ED can alter the role of the emergency clinician. Multiple studies show that implementing CDSTs based on AI is required to deliver healthcare to a growing and aging population. In conclusion, most of the literature developed prediction or identification tools in the form of clinical decision support. Although some of the literature concludes that such tools can outperform clinicians, chances are small they will be used to replace clinicians in the near future.

## Supplementary Information

**Additional file 1.** Number of articles retrieved from backtrack-search (after removing duplicates).

**Additional file 2.** Color-coding scheme of abstract search.

**Additional file 3.** Quality Assessment of the Full-Text Scan.

**Additional file 4.** Key Findings from Literature.

## Data Availability

The data are included in the additional files.
