# Supplementary material for: Influence of artificial intelligence on the work design of emergency department clinicians a systematic literature review
Source: BMC Health Serv Res. 2022 May 18;22:669. doi: 10.1186/s12913-022-08070-7 (PMC9118875; doi:10.1186/s12913-022-08070-7)
Supplement: Supplementary file 1 — Additional file 1. Number of articles retrieved from backtrack-search (after removing duplicates). [file 12913_2022_8070_MOESM1_ESM.docx]

# Additional File I

## Number of articles retrieved from backtrack-search

(after removing duplicates)

| Article | Nr. of References retrieved |
| --- | --- |
| Berlyand, Y., Raja, A. S., Dorner, S. C., Prabhakar, A. M., Sonis, J. D., Gottumukkala, R. V., Succi, M. D., & Yun, B. J. 2018. How Artificial Intelligence Could Transform Emergency Department Operations. ***The American Journal of Emergency Medicine*,** *36*(8), 1515-1517. | 5 |
| Liu, N., Zhang, Z., Ho, A. W., & Ong, M. E. H. 2018. Artificial Intelligence in Emergency Medicine. ***Journal of Emergency Critical Care Medicine*,** *2*(4), 82-82. | 9 |
| Shafaf, N., & Malek, H. 2019. Applications of Machine Learning Approaches in Emergency Medicine; a Review Article. ***Archives of Academic Emergency Medicine,****7*(1). | 17 |
| Stewart, J., Sprivulis, P., & Dwivedi, G. 2018. Artificial Intelligence and Machine Learning in Emergency Medicine. ***Emergency Medicine Australasia***, *30*(6), 870-874. | 9 |
