# Supplementary material for: Influence of artificial intelligence on the work design of emergency department clinicians a systematic literature review
Source: BMC Health Serv Res. 2022 May 18;22:669. doi: 10.1186/s12913-022-08070-7 (PMC9118875; doi:10.1186/s12913-022-08070-7)
Supplement: Supplementary file 2 — Additional file 2. Color-coding scheme of abstract search. [file 12913_2022_8070_MOESM2_ESM.docx]

# Additional File II

## Color-coding scheme of abstract search

*The articles are numbered in the order that they were found during the literature search

| Color | Theme |
| --- | --- |
|  | Prediction risk for heart failure/cardiac arrest/chest pain |
|  | Prediction hospitalization need/critical care |
|  | Prediction/(Early) identification of acute kidney injury/failure |
|  | Prediction/(Early) identification of sepsis |
|  | Prediction of waiting time |
|  | Assisting clinical decision support/management/influence on job design |
|  | Physician vs. AI |
|  | Triage-classification |
|  | Machine learning is not superior technique/clinical utility is not sure |
|  | Prediction in general |
|  | Identification in general |

| Reason for exclusion | Number |
| --- | --- |
| Pediatric theme |  |
| Review |  |
| Type of ML/AI is too complicated |  |
| Misleading title |  |
| COVID-19 theme |  |
| Not really AI |  |
| Not about ED |  |
|  |  |

| Nr. | Removed | Color | | | | | | Prediction/Identification | | | | Main theme |
| --- | --- | --- | --- | --- | --- | --- | --- | --- | --- | --- | --- | --- |
|  |  |  | |  | |  | |  | | | | Trauma injury severity prediction (thorax) |
|  |  |  | |  | |  | |  | | | | Prediction of heart failure risk |
|  |  |  | |  | |  | |  | | | | Prediction of hospital demand |
|  |  |  | |  | |  | |  | | | | Early identification of acute kidney failure |
|  |  |  | |  | |  | |  | | | | Prediction of major adverse cardiac events |
|  |  |  | |  | |  | |  | | | | Prediction of 30-day mortality in pneumonia patients |
|  |  |  | |  | |  | |  | | | | Early detection of risk for cardiac arrest |
|  |  |  | |  | |  | |  | | | | Prediction of in-patient admission |
|  | 1-X |  | |  | |  | |  | | | | Predicting need for hospitalization pediatric asthma |
|  |  |  | |  | |  | |  | | | | Predicting need for hospitalization/critical care asthma |
|  |  |  | |  | |  | |  | | | | Feasibility of automating CDSSs in EHR |
|  |  |  | |  | |  | |  | | | | Improving ED triage/patient classification |
|  |  |  | |  | |  | |  | | | | Application of AI in ED triage acute abdominal pain |
|  | 2-X |  | |  | |  | |  | | | | Review of Stewart et al. used for backtracking |
|  | 3-X |  | |  | |  | |  | | | | Early identification of sepsis in ED |
|  | 2-X |  | |  | |  | |  | | | | Forecasting medical patient LOS at presentation in ED |
|  |  |  | |  | |  | |  | | | | Early identification of acute kidney injury |
|  |  |  | |  | |  | |  | | | | Prediction of potential ED bounce-backs |
|  |  |  | |  | |  | |  | | | | Detection of delayed septic shock |
|  | 1-2-X |  | |  | |  | |  | | | | Follow-up on outcome prediction in pediatric ED |
|  |  |  | |  | |  | |  | | | | Prediction of early mortality in ED |
|  | 2-X |  | |  | |  | |  | | | | Guide to deep learning in healthcare |
|  |  |  | |  | |  | |  | | | | Prediction of hospitalization need from ED |
|  | 4-7-X |  | |  | |  | |  | | | | Detection of altered mental status |
|  | 2-X |  | |  | |  | |  | | | | Big data in medicine |
|  |  |  | |  | |  | |  | | | | Classification of hospital admission |
|  |  |  | |  | |  | |  | | | | Identification of suspected infection |
|  |  |  | |  | |  | |  | | | | Linking algorithm 🡪 can alter management |
|  | 2-X |  | |  | |  | |  | | | | Review of nr. 30 |
|  |  |  | |  | |  | |  | | | | ED triage prediction of clinical outcomes |
|  |  |  | |  | |  | |  | | | | Detection/identification of septic shock |
|  |  |  | |  | |  | |  | | | | Detection/identification of high-risk septic patients |
|  | 2-X |  | |  | |  | |  | | | | Review of Berlyand et al. used for backtracking |
|  |  |  | |  | |  | |  | | | | Improving documentation of presenting problems |
|  |  |  | |  | |  | |  | | | | Prediction of clinical outcomes in ED triage |
|  |  |  | |  | |  | |  | | | | Detection of chest pain 🡪 myocardial infarction |
|  |  |  | |  | |  | |  | | | | Prediction of septic shock in ED triage |
|  | 2-X |  | |  | |  | |  | | | | Prospects and pitfalls of ML in clinical practice |
|  | 4-7**~** |  | |  | |  | |  | | | | Prediction of postoperative hospital readmission |
|  | 2-X |  | |  | |  | |  | |  | | Prediction/detection of adults vulner. for adverse drug events |
|  | 2-X |  | |  | |  | |  | | | | Systematic review of ML vs usual care in ED |
|  |  |  | |  | |  | |  | | | | Identification of high-risk patients in ED triage |
|  | 4-X |  | |  | |  | |  | | | | Improve diagnostic accuracy of septic arthritis in the knee |
|  |  |  | |  | |  | |  | | | | Prediction of workload per patient |
|  |  |  | |  | |  | |  | | | | Prediction of clinical orders during triage |
|  |  |  | |  | |  | |  | | | | Prediction of hospital admission during triage |
|  |  |  | |  | |  | |  | | | | Prediction of hospital admission older patients |
|  | 4-7**~** |  | |  | |  | |  | | | | Predicting ED usage + hospitalization |
|  | 4**~** |  |  | |  | |  | |  | | Prediction of need for hospitalization ICU in triage | |
|  |  |  | |  | |  | |  | | | | Prediction of UTIs |
|  |  |  | |  | |  | |  | | | | Prediction of waiting time |
|  | 1-X |  | |  | |  | |  | | | | Prediction of admission in pediatric ED |
|  | 2-X |  | |  | |  | |  | | | | Prediction of emergency department patient disposition |
|  | 7**~** |  | |  | |  | |  | | | | Prediction of opioid administration |
|  |  |  | |  | |  | |  | | | | Identify need for CT scans during triage |
|  |  |  | |  | |  | |  | | | | Prediction of risk composite critical outcomes in triage |
|  | 2-X |  | |  | |  | |  | | | | Reduce waiting times in ED |
|  | 2-X |  | |  | |  | |  | | | | Ten ways artificial intelligence will transform primary care |
|  | 7**~** |  | |  | |  | |  | | | | Identify fall-risk in older adults after ED visit |
|  | 4-X |  | |  | |  | |  | | | | Racial disparities in asthma ED visits |
|  |  |  | |  | |  | |  | | | | Identification of “frequent flyers” |
|  | 3**~** |  | |  | |  | |  | | | | Prediction of risk-stratification for chest pain |
|  |  |  | |  | |  | |  | | | | Prediction of the need for critical care EMSs in triage |
|  |  |  | |  | |  | |  | | | | Prediction of hospitalization. Risk-stratification |
|  | 2-X |  | |  | |  | |  | | | | AI in emergency medicine |
|  |  |  | |  | |  | |  | | | | Identification of seizures in triage |
|  | 2-5-X |  | |  | |  | |  | | | | AI education in radiology |
|  | 5**~** |  | |  | |  | |  | | | | Radiographic diagnosis of COVID-19 pneumonia in ED |
|  | 4-X |  | |  | |  | |  | | | | Virtual reality to assess emergency medicine learners’ competence |
|  | 2-X |  | |  | |  | |  | | | | Overview of ML in relation to emergency medicine |
|  |  |  | |  | |  | |  | | | | Identifying scaphoid fractures. |
|  | 2-X |  | |  | |  | |  | | | | Overview of ML in relation to emergency medicine. Same as nr. 70 |
|  | 2-X |  | |  | |  | |  | | | | Same as nr. 70/72 |
|  |  |  | |  | |  | |  | | | | Identifying abnormalities in ECG’s |
|  | 6**~** |  | |  | |  | |  | | | | Voice-based training for emergency care provider |
|  |  |  | |  | |  | |  | | | | Prediction of disposition based on ED triage notes |
|  |  |  | |  | |  | |  | | | | Prediction of waiting time |
|  |  |  | |  | |  | |  | | | | Prediction of progression to delayed septic shock |
|  | 2**~** |  | |  | |  | |  | | | | Detection of strokes (practical framework) |
|  | 2-X |  | |  | |  | |  | | | | Review of Shafaf et al. used for backtracking |
|  | 2-X |  | |  | |  | |  | | | | Prediction model not tested |
|  | 4-7-X |  | |  | |  | |  | | | | Streamlining pre- and intra-hospital care for severe trauma patients |
|  | 2-X |  | |  | |  | |  | | | | Predicting cardiac arrest using deep learning |
|  | 7**~** |  | |  | |  | |  | | | | ML on dispatcher recognition of cardiac arrest |
|  | 6**~** |  | |  | |  | |  | | | | Discussion of diagnostic uncertainty |
|  | 7-X |  | |  | |  | |  | | | | Prediction of acute kidney injury |
|  | 7**~** |  | |  | |  | |  | | | | Development of acute kidney injury prediction model |
|  | 7-X |  | |  | |  | |  | | | | Prediction of ICU readmission |
|  | 7**~** |  | |  | |  | |  | | | | Predictive modeling in urgent care |
|  | 7**~** |  | |  | |  | |  | | | | Detection of pneumonia via x-ray |
|  | 7**~** |  | |  | |  | |  | | | | Prediction of aneurysm rupture risk |
|  | 2-7-X |  | |  | |  | |  | | | | DL with EHRs |
|  | 7-X |  | |  | |  | |  | | | | Classifying radiology free-text reports |
|  | 7-X |  | |  | |  | |  | | | | Detection algorithm CT examinations |
|  | 7**~** |  | |  | |  | |  | | | | Analyzing orthopedic trauma |
