# Supplementary material for: Influence of artificial intelligence on the work design of emergency department clinicians a systematic literature review
Source: BMC Health Serv Res. 2022 May 18;22:669. doi: 10.1186/s12913-022-08070-7 (PMC9118875; doi:10.1186/s12913-022-08070-7)
Supplement: Supplementary file 3 — Additional file 3. Quality Assessment of the Full-Text Scan. [file 12913_2022_8070_MOESM3_ESM.docx]

# Additional File III

## Quality Assessment of the Full-Text Scan

| Color | Reason |
| --- | --- |
|  | Could only find an abstract |
|  | Not about the ED |
|  | Kind of AI is not useful in clinical setting |

| **Nr.** | **Author** | **Mentions influence** | **Replacing of doctor** | **Motivation** |
| --- | --- | --- | --- | --- |
| 1 | Kulshrestha et al. | NO |  |  |
| 2 | Sax et al. |  |  | Reduce hospital admission |
| 3 | Vollmer et al. |  |  | Improve healthcare delivery |
| 4 | Martinez et al. |  |  | Identify acute kidney injury |
| 5 | Zhang et al. |  |  | Improve ED decision making |
| 6 | ~~Kang et al.~~ |  |  | = |
| 7 | Jang et al. |  |  | Reduce overcrowded EDs |
| 8 | Rendell et al. |  |  | Improve clinical care/predict admission |
| 10 | Goto et al. |  |  | Reduce mortality/morbidity (asthma) |
| 11 | ~~Perry et al.~~ |  |  |  |
| 12 | Levin et al. |  |  | Improve ED triage |
| 13 | Farahmand et al. |  | YES | Improve ED triage |
| 17 | ~~Hinson et al.~~ |  |  |  |
| 18 | ~~Peng et al.~~ |  |  |  |
| 19 | ~~Wardi et al.~~ |  |  |  |
| 21 | Klug et al. |  |  | Improve categorization/ED triage |
| 23 | ~~Frisch et al.~~ |  |  |  |
| 27 | Horng et al. |  |  | Reduce overcrowded EDs/identify sepsis |
| 28 | Redfield et al. |  |  | Improve healthcare delivery |
| 30 | Raita et al. |  |  | Reduce overcrowded EDs/predict clinical outcome |
| 31 | Yun et al. |  |  | Reduce mortality (septic shock) |
| 32 | Chiew et al. |  |  | Reduce mortality (septic shock) |
| 34 | Greenbaum et al. |  |  | Improve ED decision making |
| 35 | Yu et al. |  |  | Reduce overcrowded EDs/Improve ED triage |
| 36 | ~~Lindholm et al.~~ |  |  |  |
| 37 | Kim et al. |  |  | Identify sepsis |
| 42 | Jiang et al. |  |  | Improve ED triage (CVD) |
| 44 | Wang et al. |  |  | Predict ED Physician workload |
| 45 | Hunter-Zinck et al. |  |  | Reduce LOS (anticipate orders) |
| 46 | Hong et al. |  |  | Reduce overcrowded EDs/predict hospitalization need |
| 47 | Mowbray et al. |  |  | Predict patient disposition (elderly) |
| 50 | Taylor et al. | NO |  | Reduce overcrowded EDs/predict UTI |
| 51 | Pak et al. |  |  | Increase patient satisfaction/reduce patient waiting times |
| 55 | Klang et al. |  |  | Optimize ED patients’ throughput |
| 56 | Fernandes et al. |  |  | Identify high mortality risk |
| 61 | Frost et al. |  |  | Reduce ED visits |
| 63 | ~~Kang et al.~~ |  |  |  |
| 64 | Falavigna et al. |  |  | Improve ED decision making |
| 66 | ~~Parvizi et al.~~ |  |  |  |
| 71 | Ozkaya et al. |  | YES | Increase diagnostic performance (scaphoid fracture) |
| 74 | Smith et al. |  |  | Comparing AI vs. Conventional computer |
| 76 | Tahayori et al. |  |  | Predict patient disposition (ED triage) |
| 77 | Kuo et al. |  |  | Reduce overcrowded EDs/predict patient waiting times |
| 78 | ~~Wardi et al.~~ |  |  |  |
