# Supplementary material for: Influence of artificial intelligence on the work design of emergency department clinicians a systematic literature review
Source: BMC Health Serv Res. 2022 May 18;22:669. doi: 10.1186/s12913-022-08070-7 (PMC9118875; doi:10.1186/s12913-022-08070-7)
Supplement: Supplementary file 4 — Additional file 4. Key Findings from Literature. [file 12913_2022_8070_MOESM4_ESM.docx]

# Additional File IV

## Key Findings from Literature

| nr. | | Author, Year &  Country | | Participants | | Kind of data used | | Global Aim | | Focused Aim | | Central Aim | | Key Findings |
| --- | --- | --- | --- | --- | --- | --- | --- | --- | --- | --- | --- | --- | --- | --- |
| 11. | | Rendell et al. (2019)  Australia | | 1,721,294 | | Data routinely available at triage | | To reduce cognitive load on clinicians | | To accurately predict future outcomes | | To predict risk for admission | | - It is a clinical decision support tool  - EDs can use ML to act more proactively, e.g., plan and arrange required in-patient beds and resources.  - ML will most likely become very important to ED clinicians as the amount of data they work with is increasing while they still need to be able to act on all that information rapidly  - ML can influence how future healthcare is provided  - Improves patient flow  - Tool can assist clinicians by determining the patient disposition  - There are no studies that assess the influence of ML clinical decision support tools on ED clinician behavior and patient flow  - It is a challenge for ED clinicians to implement the use of this data in clinical practice  - It can alert clinicians  - It is institutional specific  - Cannot replace doctors. They can minimalize variability and mistakes (434) |
| 15. | Chiew et al. (2019)  Singapore | | 214 | | Data routinely available at triage and from the EHR | | To reduce 30-day mortality | | To accurately predict/identify future outcomes | | To predict risk for sepsis | | - Concerns about a prediction model  - Early identification of high-risk septic patients in the ED can serve as a guideline for appropriate management and disposition. Consequently, it can improve outcomes  - Due to the implementation of EHRs and widely available data, ML has become favored in modeling patient health status  - Usage of easily obtainable clinical information during triage and without chart review  - Usage of this kind of clinical information facilitates early recognition of high-risk patients and, therefore also the appropriate care resources needed  - Used 30-day mortality as this is sepsis-related and more meaningful for physicians regarding the administering of potential interventions  - *Might* not be generalizable (possibly due to the small sample size and single-center focus)  - There is no current gold standard for determining septic patients  - Multiple EDs use electronic data collection systems, making it increasingly feasible to employ ML models.  - Can be used as CDST for early and appropriate management | |
| 16. | | Falavigna et al. (2019)  Italy | | 1,804 | | Data routinely collected at triage | | To help physicians (increase with efficiency (costs) and effectiveness of treatment) | | To accurately predict future outcomes | | To predict the need for hospitalization | | - Physicians and doctors might want different things, but sensitivity should be the main aim of a stratification rule because only then it correctly supports the decision-making process in EDs.  -Using mobile applications can tailor risk stratification according to a patient-specific case and the decision-making process  A decision-making process supported by AI can improve the healthcare sector, alter management, and enhance the rate of syncope patients' admission. This can then guarantee that resources are used more effectively, and it can reduce the cost of unnecessary treatments and hospitalization  specific  - It cannot replace physicians  - Needs external validation |
| 17. | | Farahmand et al. (2017)  Iran | | 215 | | Data routinely available at triage. | | To streamline crowded EDs with improving triage | | To develop an AI tool that could take away the need for an expert emergency medicine physician at triage level 4 (ESI-4). | | to estimate ESI-4 score of patients presenting with acute abdominal pain with AI-based tools and without the estimate of the required resources. | | - Gold standard was ESI level (e.g., level of triage) indicated by the physician  - the predictions that were made by the AI-based tool were compared to the physician (gold standard)  - A triage model that is AI-based could speed up the decision-making process in an overcrowded ED while being reproducible and measurable. Especially levels 3-5, which are the most difficult levels to estimate (by triage nurses)  - It has the ability to replace an expert physician partially. |
| 18. | | Fernandes et al. (2020)  Portugal | | 235,826 | | Data routinely collected at triage + a number of other variables | | To enhance ED triage systems | | To accurately predict future outcomes | | Risk prediction of mortality and cardiac arrest. | | - Study mentions that although both are undesirable, it is better to overestimate than to underestimate the chances of a combined outcome for a group of patients at high risk.  - This study aims to enhance the existing triage model by adding ML and to prevent under-triaging then |
| 19. | | Frost et al. (2017)  Canada | | 101,174 | | Data available in the cumulative patient profile of family physicians’ electronic medical records. | | To prevent overcrowding of EDs + limit costs | | To accurately predict future outcomes | | To predict future emergency department visits and high overall system costs. | | - Article mentions that the method described can have consequences for the emergency clinician.  - Mentions implications for clinicians: it is plausible that a tool can use the text from EMRs to create a list of the patients who are inclined to become high users or cause high costs  - When clinicians or other staff are alerted to patients who are most likely to become high users or cause high costs, corrective action can be started to the advantage of the patient and the healthcare delivery system  - It could possibly advance patient care and lessen costs significantly  - Generalizability *might* be limited |
| 20. | | Goto et al. (2018)  USA | | 3206 | | Data routinely collected at triage + a priori knowledge | | To reduce ED morbidity and mortality | | To improve ED triage | | to predict the disposition of asthma and COPD exacerbation patients after ED triage | | - At ED triage, there is the chance to identify and separate the critically ill from the stable patients.  - ESI (the currently used method) relies profoundly on subjective factors, such as provider judgment. The use varies between operators.  - Predictors could improve but not make perfect. ED disposition decisions remain difficult.  - It can be used as an assistive technology  As a result, it can improve the clinician's ED triage decision-making.  - Can eventually lead to more accurate clinical practice in ED.  - Can also lead to more efficient clinical practice |
| 21. | | Greenbaum et al. (2019)  USA | | 279,231 | | Data routinely collected as part of clinical care (independent of the project) | | Increase physician satisfaction, decrease physician burn-out | | To optimize EHR workflows | | To improve the efficiency and quality of structured data | | - Improved quality of documentation  - Reduce the burden on triage nurses  - Saves 88h on documentation per year  - Structured data is more valuable than unstructured data  - Reduced workload  - Could be used for other areas than presenting problems, such as diagnosis, procedures, and problem lists.  - Outcomes *might* not be generalizable |
| 22. | | Hong et al. (2018)  USA | | 560,486 | | Data routinely collected at triage + Data collected from previous ED visits (focus) | | To reduce ED crowding + to improve patient care + | | To accurately predict future outcomes | | To predict hospitalization or discharge at ED triage | | - Offers a clinical decision support tool  The article mentions that prediction models in healthcare are often used to increase the quality of care and increase logistical efficiency.  - Early identification of patients in need of hospital admission may increase the patient outcomes  - Study mentions explicitly that it cannot address the correctness of individual clinical decision making  - Possible implementation barrier |
| 23. | | Horng et al. (2017)  Australia | | 230,936 | | Data routinely collected at triage | | To reduce mortality and morbidity, and costs | | to accurately predict future outcomes | | To predict the risk for sepsis at ED triage + to implement early protocolized care | | - Improves clinical decision support  - Mentions that clinical support systems have been proven to be able to guide physicians through predefined workflows.  - The study considers that implementing a trigger for the risk of sepsis cannot depend on the triage nurse.  - Method used does not entail that any additional work will be demanded from the triage nurse.  - Method used is easily generalizable to other kinds of clinical decision support that use data available at ED triage.  - Method also focuses on creating a prediction model based on unstructured data instead of structured data  - It is important that clinical decision support in the ED is timely, as timeliness is crucial in an ED.  - The need for clinical decision support systems will increase  - If not used correctly, it has the potential to interrupt the workflow  - In need of external validation |
| 24. | | Hunter-Zinck et al. (2019)  USA | | 140,855 | | Data routinely collected at triage + medical history from EHR | | To reduce length of stay (LOS) in ED | | To accurately predict future outcomes | | To predict clinical ordering at ED triage | | - Article mentions that clinical support tools can enhance efficiency and decrease differences in decision making concerning resource distribution and patient trajectories  - Clinical decision support tools for predicting clinical ordering can make healthcare providers shift from a reactive to a proactive resource distribution  - Predictive model used in the study could be used for data-driven ordering sets  - LOS can be reduced (consequences!)  - Generalizability needs to be explored  - Model can be retrained |
| 25. | | Jang et al. (2020)  Republic of Korea | | 374,605 | | Data routinely collected at triage | | To better cope with overcrowding EDs | | To accurately predict future outcomes | | To predict cardiac arrests in the ED | | Implementing predictive models for cardiac arrest can help clinicians detect adverse events earlier. This way, they can take preventive measures  - These models can also reduce alarm fatigue and desensitization for alarms because the number of false alarms would decrease. This could then improve healthcare delivery in its entirety.  The reasons for predictions are not presented by the predictive models, leading to clinicians not understanding the basis for the forecast. It increases the difficulty of what course of action to take.  - it can help alert the clinician in an overcrowded ED  - could be used at triage as well as at the early warning system |
| 26. | | Jiang et al. (2021)  Hong Kong, China | | 17,661 | | Data routinely collected and available at triage | | To reduce mortality and morbidity + to reduce overcrowding EDs | | To accurately predict future outcomes | | To predict triage levels for patients with suspected CVD | | - Explains e-triage  - E-triage can be used to advance patient risk management and decision-making in the ED.  - Machine learning for clinical decision support tools can allow clinicians to refocus on clinical care again because it reduces the cognitive burden that has been increasing  - Models used in the study can be used as adjunct tools for triage nurses  - Survey under nurses showed there is a request for automatic severity calculators  - A clinical decision support tool for triage may reduce errors  - Tool can be altered for use at home to help decide whether even to visit the ED  - Might not be generalizable |
| 27. | | Kim et al. (2020)  Republic of Korea | | 49,299 | | Data routinely available at triage + laboratory results and clinical findings | | To cope with the increasing demand for clinical care in EDs | | To accurately predict future outcomes | | To predict septic shock at ED triage | | - It is not easy to detect sepsis already at ED triage  - There is an increasing demand for critical care in EDs, which makes the allocation of resources difficult  - A sepsis screening tool might help physicians allocate their resources to the patient with a severe illnesses such as septic shock  - *Might* not be generalizable |
| 28. | | Klang et al. (2020)  Israel | | 561,933 | | Data available at triage | | To alleviate overburdened EDs and increase patient’s throughput | | To accurately predict and identify future outcomes | | To identify patients in need of a head CT scan at triage | | - Usually, the ED physician is responsible for deciding when a head CT scan is necessary  - Can be used as a clinical decision support tool  - Workload of ED and physicians is rising while it is physically impossible to keep up with the demand. AI is needed to alleviate this problem  - This clinical decision support tool can enhance hospital efficiency and improve patient care because patients’ throughput will increase  Using this tool, there is an ability to diagnose patients at an earlier stage, leading to faster treatment and shorter ED visits.  - The tool can be used as an alert to make decisions formerly made by physicians  - Generalizability is not sure, but probably possible |
| 29. | | Klug et al. (2019)  Israel | | 799,522 | | Data collected and available at triage (from EMR) | | To alleviate overburdened EDs by improving patient categorization | | To accurately predict future outcomes | | To predict ED mortality | | - Can be used as a clinical decision tool for clinicians to accelerate treatment and to identify patients at high risk for mortality and who were under-triaged.  - The use of a simplified model with a few variables makes it easier to understand what variables are driving the clinical decision support tool.  - Study serves as evidence that AI-based clinical decision support tools can enhance triaging  - It is a *tool,* and it does not aim to substitute clinical judgment  - The tool can make clinical decision making more consistent and reduces the dangers of over-or under-triaging  - Generalizability might be limited |
| 30. | | Kulsrestha et al. (2021)  USA | | 6,891 | | Data routinely available at triage (Unstructured data from the EHR) | | To improve patients’ throughput | | To accurately predict and identify future outcomes | | To identify severe chest (thorax) injury | | - This classifier tool makes data sharing between hospitals easier as everything is coded  - This classifier tool makes it possible for hospitals to engage in improving the quality of trauma care and clinical trials  - Enables a better oversight for non-trauma centers concerning care for patients with a chest injury  - Implementation barrier (external validation needed) |
| 31. | | Kuo et al. (2020)  Hong Kong, China | | 11,690 | | Data containing records of patient visits + knowledge of ED system | | To reduce ED overcrowding | | To accurately predict future outcomes | | Predict patient waiting times | | - Patients can adjust their decision to visit an ED for non-urgent care accordingly  - Hospital can better allocate their resources to prevent possible overcrowding  A hospital might act proactively on a long waiting time by paging the doctors on call in a timely manner.  - It could improve patient satisfaction but also overall satisfaction  - It can improve staffing decisions |
| 32. | | Levin et al. (2018)  USA | | 172,726 | | Data routinely collected at triage + medical history from EHR | | To reduce ED overcrowding | | To improve ED triage | | To develop an e-triage system | | - Triage sets the course for ED care as it is the first moment at which patients can be categorized  - Triage done wrong can have severe implications for the critically ill  - May be of use in EDs that are crowded with low acuity patients and are therefore challenged with managing the high numbers of these patients  - E-triage is not suitable for every patient's pathway  - When care is accelerated, it can enhance patient outcomes  - E-triage is locally bound in some areas  - E-triage is not designed to work individually rather, it can be used as a clinical decision support tool  - Stimulates triage nurse decision making to create an alliance |
| 33. | | Martinez et al. (2020)  USA | | 91,258 | | Data routinely available from the EHR at early stage (incl. lab results) | | To improve patient outcomes + to reduce adverse outcomes | | To accurately identify future outcomes early | | To identify patients at risk for acute kidney failure | | - It is a clinical decision support tool  - Data used was judged by clinicians, amongst others  - In need of external validation (implementation barrier)  - Advantage of ML is that it can be adapted to different populations  - The main objective is to inform decision support  - Clinical decision support is **essential** because clinicians treat an increasing number of patients inside the hospital and an even more significant number outside the hospital.  - Needs external validation |
| 34. | | Mowbray et al. (2020).  Canada | | 2,274 | | Data with a comprehensive set of geriatric syndromes (not typically available) | | To prevent adverse outcomes | | To accurately predict future outcomes | | To predict/identify geriatric need for hospitalization | | - Deciding on elderly patient disposition proves to be difficult for clinicians  - Timely identification of patients in need of hospital admission can speed up admission and treatment  - Healthcare providers can work proactively instead of reactively  - Timely identification of hospitalization needs in the ED can influence the prediction of bed capacity needs and can reduce LOS  - Timely identification of hospitalization needs can also accelerate in-patient care plans, consultations, and admissions  - Proactive discharge planning  - Generalizability might be limited |
| 35. | | Ozkaya et al. (2020).  Turkey | | 390 | | Radiographic images | | To improve patient outcomes | | To accurately identify outcomes + reduce need for physician | | To identify wrist (scaphoid) fractures | | - a method that can be of use to less experienced doctors in an ED setting  - especially useful for EDs of hospitals that do not have experienced hand surgeons  - compares AI with a physician  - AI does not perform experienced orthopedic specialists, is similar to less experienced orthopedic specialists, and is better than ED physician  - will probably outperform in the future. Can be trained faster than the doctor (probably) |
| 36. | | Pak et al. (2021)  Australia | | 94,284 | | Routinely collected administrative data | | To improve patient outcomes (and satisfaction) | | To accurately predict future outcomes | | To predict patient waiting time | | - Knowledge of waiting time can decrease anxiety and the number that leave without being seen  - Works best during the day  - External validation is limited but is doable  - When hospitals place their waiting times, e.g., online, patients can adjust their decision on coming to the ED, leading to a better distribution of patients  - External validity might be limited |
| 37. | | Raita et al. (2019)  USA | | 135,470 | | Data routinely available at triage | | To cope with overcrowded EDs | | To accurately predict future outcomes | | To predict critical care and hospitalization outcomes at ED triage | | - Helps to allocate ED resources better and to provide urgent care timely  ED triage goals are to accurately categorize patients into acuity levels, ranging from critical to non-urgent care.  - ESI is mostly subjective, causing a significant difference in use between healthcare providers  - Can be used as clinical decision support  - ML methods are crucial to enhance future clinical decision support technology  - Improves patient care and hospital resource allocation  - Needs external validation  - Generalizability is probably possible |
| 38. | | Redfield et al. (2020)  USA | | 14,032 | | Age, gender, first and last name, SSN, and DOB | | To improve patient outcomes | | To accurately link future outcomes | | To link prehospital records to hospital records | | - Linkage of electronic patient care reports (ePCRs) and EMRs can alter management in the ED  - Record linkage can increase the quality of care and education for healthcare providers (both EMS and ED)  - Can reduce mortality in trauma patients  - Can be used without a human in the loop (only for false negatives). So, it does not add additional work to ED personnel  - Not externally validated, limits the generalizability |
| 39. | | Sax et al. (2021)  USA | | 26,189 | | Data available within 3 hours of ED arrival | | To (safely) reduce hospital admissions | | To accurately predict future outcomes | | To predict risk for a 30-day serious adverse event | | - Generalizability may be limited because not all hospitals are equipped with the right resources.  - Not yet compared to physician gestalt, future studies must address the prediction ability of a model in combination with a physician.  - Can be implemented to use as clinical decision support  - interface can run in the background while the physician determines patient disposition. A physician can then discuss the risk prediction with the patient to agree on the right disposition and how to manage  - It would lead to an increase in grounded decision making; it would improve the use of resources and patient care for high-risk patients.  - Can promote shared decision making  - Not externally validated (was not the aim either) |
| 40 | | Smith et al. (2019)  USA | | 1473 | | ECG recordings evaluated by expert cardiologists | | To improve patient outcomes and improve physician ability | | To accurately identify outcomes | | To identify ECG outcomes | | - Accurate automated ECG interpretation tools can improve patient management.  - Deep learning keeps learning all the time  - DNN was not compared to a gold standard, e.g., an expert ECG interpreter  - Tool can warn the clinician when it recognizes abnormalities that are hard to detect to help the clinician act appropriately |
| 41. | | Tahayori et al. (2020)  Australia | | 249,532 | | Data from triage notes | | To increase flow in crowded EDs | | To accurately predict future outcomes | | To predict patient disposition during ED triage | | - Aim is to develop an AI-based algorithm similar to ED physicians  - Tool was compared with human experts (without looking at vital signs) and performed better. However, humans are more sensitive  - Early identification of patients in need of admission can improve resource distribution because the flow of care is better and double handling is prevented.  - Securing a bed for a patient is done earlier, which leaves more time for allocation  - Tool can protect against falsely early discharge and reduce excess re-admission costs and use of hospital resources.  - Needs multicenter validation + re-training |
| 42. | | Taylor et al. (2018)  USA | | 80,387 | | Data available during ED visit until admission (structured) | | To reduce diagnostic errors (and costs + overutilization of resources) | | To accurately predict/identify future outcomes | | To predict/identify UTIs at an early stage | | - Clinical decision support tool  - Misdiagnosing UTIs leads to increased costs, overutilization of resources, and resistance to antibiotics  - Best performing ML-based tool was compared to clinical judgment  - Could be used in an app, assuming it would not add to the already heavy workload of an ED clinician  - Requires external validation |
| 43. | | Vollmer et al. (2021)  UK | | 730 days | | Data on daily attendances, weather, holidays, flu season, and known events causing high demand | | To improve healthcare delivery | | To accurately predict future outcomes | | To predict future hospital demand | | - Mentions overcrowded EDs as a significant issue  - Also mentions that staffing right is an issue  - Model can be retrained regularly and efficiently  - Training of the model can be done in just minutes  - Model can help with staffing decisions, such as adjusting schedules and arranging for additional demand  - Can help EDs work as efficiently as possible  - Future prospect is to be more flexible at rostering and communicate innovatively with the local society. |
| 44. | | Wang et al. (2021)  USA | | 5,427 | | Data available from the EHR (1 hr, 2hrs, whole visit) | | To improve healthcare provider wellbeing and preserving patient safety | | To accurately predict future outcomes | | To predict clinician workload | | - It is a prediction tool that predicts patient-related workload. This workload can then be linked to the clinician's workload.  - When clinician workload is known and assigned to clinicians, it can be used in a management device or model.  - The information on workload can be taken into account when clinicians are assigned new patients  - Can also be used to recognize excessive clinician workload  - When comparing proxy workload with the actual workload can increase the burden on clinicians |
| 45. | | Yu et al. (2020)  Republic of Korea | | 86,309 | | First data collected at arrival | | To cope with increasing ED load | | To accurately predict future outcomes | | To predict adverse clinical outcomes at ED triage | | - Designed as clinical decision support  - Model was not designed to replace clinicians but merely as an assistant tool that can help make decisions while clinicians assess patients  - Reduces dependence on subjective judgment  - In need of external validation |
| 46. | | Yun et al. (2021)  Republic of Korea | | 41,687 | | Data routinely collected at triage | | To improve patient outcomes (incl. mortality) | | To accurately identify outcomes | | To identify septic shock within 24h after ED arrival | | - Used for patients with already suspected infection, which should be established first by physicians  - Designed as clinical decision support, as an early cautioning system for septic shock  - This could reduce ICU admission and mortality  - In need of external validation |
| 47. | | Zhang et al. (2020)  Taiwan | | 85,254 | | Data not all routinely collected at triage (long list) | | To reduce diagnostic errors and excess costs | | To accurately predict and identify outcomes | | To predict and identify serious cardiac events | | - Designed together with a group of physicians, nurses, data scientists to establish clinical utility (assumption)  - Designed as clinical decision support to assist physicians in real-time  - Physician can push the AI button for assistance  - Patient disposition with chest pain as the chief complaint is always tricky, especially when EDs are overcrowded  - Delivers ED physicians with faith in enhancing patient outcomes  - New legal doctrine is necessary with the improvement of technology because it brings forward many new consequences and problems in this area.  - Mentions need future research of the consequences on clinical practice, e.g., decision-making, acceptance, etc.  - In need of external validation |
